# Veterinary Medicine

**Published:** 1850-05

**Authors:** 


### VETERINARY MEDICINE.

Dr. Edward Brooks, of Boston, who, three years ago, wrote for this Journal a highly interesting paper on veterinary medicine, returned, a few months ago from a protracted stay in Europe, where he devoted his time and fine talents to the study of the diseases of the horse. Dr. Brooks immediately entered upon the duties assigned him by the Legislative Agricultural Society, of Massachusetts, and has already delivered before that body, two lectures on the history and progress of veterinary medicinè. We are pleased to find them mentioned in very flattering terms, by the public prints of Boston. In one of them he announced a series of lectures, of an extended and more practical character, for next winter. We but speak from personal observation, when we say that Dr. Brooks has cultivated the subject which he proposes to teach, with the most praiseworthy diligence and industry. We believe we but express the desire of every physician and humane man in the country, when we wish Dr. Brooks's success may be equal to his merits, and that he may awaken an interest in the diseases of the horse, which shall lead to their being more generally and systematically studied.

